# The Direct Medical Costs of Sickle Cell Disease in Saudi Arabia: Insights from a Single Center Study

**DOI:** 10.3390/healthcare13040420

**Published:** 2025-02-15

**Authors:** Yazed AlRuthia

**Affiliations:** Department of Clinical Pharmacy, College of Pharmacy, King Saud University, Riyadh 11451, Saudi Arabia; yazeed@ksu.edu.sa; Tel.: +966-114677483; Fax: +966-114677480

**Keywords:** sickle cell disease, healthcare costs, blood transfusion, vaso-occlusive crisis, electronic medical record, Saudi Arabia

## Abstract

**Background**: Sickle cell disease (SCD) is a rare autosomal recessive disorder that is common in countries with consanguineous marriages. It leads to various complications, including painful episodes, infections, delayed growth, stroke, and organ damage, which contribute to high healthcare utilization and costs. In Saudi Arabia, the prevalence of SCD is notably high, largely due to the frequency of consanguineous marriages. However, there has not yet been a study estimating the direct medical costs of managing SCD based on real-world data. This study aims to assess these costs in Saudi Arabia. **Methods**: Data were collected from electronic medical records (EMRs) at a university-affiliated tertiary care center. A micro-costing approach was used to estimate the direct medical costs (e.g., laboratory tests, imaging, emergency department visits, hospitalizations, prescription medications, outpatient visits, etc.) retrospectively over a 12-month follow-up period. The baseline characteristics of the patients were presented using frequencies and percentages. The costs of different healthcare services were analyzed using means and the 95% confidence intervals. A generalized linear model (GLM) with a gamma distribution was utilized to examine the association between the overall costs and patient characteristics (e.g., age, gender, duration of illness, surgeries, blood transfusions, etc.), allowing for the estimation of the adjusted mean costs. **Results**: A total of 100 patients met the inclusion criteria and were included in the analysis. The mean age of the patients was 10.21 years (±6.87 years); 53% were male, and a substantial majority (96%) had the HbSS genotype. Sixty-one percent of the patients had undergone at least one red blood cell (RBC) exchange transfusion, while 21% had undergone surgical procedures, including tonsillectomy, splenectomy, and cholecystectomy. Additionally, 45% had experienced at least one vaso-occlusive crisis (VOC), and 59% had been hospitalized at least once in the past 12 months. Factors such as the frequency of laboratory tests and imaging studies, the length of hospital stay (LOS), the rate of emergency department (ED) visits, surgical procedures, the number of prescription medications, and the frequency of blood transfusions were all significant predictors of higher direct medical costs (*p* < 0.05). The estimated mean annual direct medical costs per patient were USD 26,626.45 (95% CI: USD 22,716.89–USD 30,536.00). After adjusting for various factors, including age, gender, duration of illness, frequency of lab and imaging tests, LOS, ED visits, surgical procedures, number of prescription medications, rates of VOCs, and RBC exchange transfusions, the adjusted mean annual direct medical cost per patient was calculated to be USD 14,604.72 (95% CI: USD 10,943.49–USD 19,525.96). **Conclusions**: The results of this study emphasize the substantial direct medical costs linked to sickle cell disease (SCD), which are greatly affected by the frequency of related complications. These insights should motivate policymakers and healthcare researchers to assess both the national direct and indirect costs associated with SCD, especially given the significant number of SCD patients in Saudi Arabia.

## 1. Introduction

Sickle cell disease (SCD) is a genetic disorder inherited in an autosomal recessive manner, marked by the creation of abnormal hemoglobin S [1]. The underlying cause of this abnormality is the homozygous inheritance of the sickle cell gene (HbS), leading to the formation of hemoglobin SS (HbS/S) [2]. These abnormal cells obstruct the blood flow in small vessels, leading to vaso-occlusion, avascular necrosis, hemolytic anemia, acute chest syndrome, pulmonary hypertension, stroke, and other serious complications [3]. Such complications result in a poor quality of life and higher mortality rates, as shown in multiple studies [4,5,6]. The prevalence and incidence of SCD vary across the world, with higher prevalence and incidence rates in Africa, the Middle East, India, and Caribbean countries. It is estimated that the incidence rates of SCD range from less than 5 to 2595 cases per 100,000 live births. Meanwhile, the prevalence rates range from less than 5 to as high as 1342 cases per 100,000 people [7]. In Saudi Arabia, SCD is prevalent in certain geographic regions, with the prevalence of SCD traits ranging from 2% to 27% and up to 2.6% of people having SCD, particularly in the Eastern region due to its high rates of consanguineous marriages [8].

SCD is a burdensome health condition associated with high direct and indirect medical costs [9,10]. In the United States, the average annual direct medical costs per patient were estimated to be USD 44,160, while the indirect costs were estimated to be USD 7619 annually [10]. Additionally, the non-elderly lifetime out-of-pocket expenditures (OOPs) attributable to SCD were estimated to be USD 44,000 according to commercial health insurance claim data from 2007 to 2018 in the United States [11]. In the Middle East, the economic burden of SCD is believed to be significant, especially in countries with relatively high prevalence rates, such as Saudi Arabia and Bahrain [12]. In a study that estimated the economic burden of SCD from payer and societal perspectives in Egypt, Bahrain, Saudi Arabia, Oman, and Lebanon based on an extensive literature review and input from 41 experts, the annual economic burden of SCD from the payer’s perspective ranged from USD 1.3 million for 1000 patients in Egypt to USD 395 million for 94,455 patients in Saudi Arabia. Emergency department visits and inpatient and outpatient services contributed to more than 50% of the total direct medical costs, which ranged between USD 5126 in Egypt and USD 29,654 in Lebanon per patient per year among patients with five or more VOCs per year. On the other hand, the annual societal burden of SCD ranged from USD 1.79 million in Egypt to USD 1.4 billion in Saudi Arabia, resulting in annual societal costs per patient of USD 1749 and USD 15,284.35 in Egypt and Saudi Arabia, respectively [13]. The economic burden of SCD was further examined in Saudi Arabia using validated patient-reported measures to estimate both the direct and indirect costs among 217 adult SCD patients (≥18 yrs.) by Shdaifat et al. in the Eastern region. It was found that the average annual per-patient costs were estimated to be USD 48,506 and USD 21,415 from societal and payer perspectives, respectively [14].

Previous studies have made attempts to quantify the direct medical costs associated with sickle cell disease (SCD) in Saudi Arabia; however, these efforts have often been limited by a lack of real-world data. As a result, they have not accurately captured the comprehensive financial impact of managing SCD within the region. This study is crucial, as it seeks to provide a detailed and precise estimate of the annual direct medical costs related to SCD management. By focusing on the perspective of public payers in Saudi Arabia, this research aims to illuminate the intricate financial demands of SCD treatment and highlight the implications for healthcare planning, resource allocation, and budget management. Through a thorough analysis of the treatment modalities, hospitalization rates, outpatient services, and medication costs, this study aspires to fill the existing knowledge gap and aid policymakers in making informed decisions regarding the allocation of healthcare resources and the formulation of effective strategies for SCD management in this country.

## 2. Materials and Methods

### 2.1. The Study Design and Patient Analysis

In this single-center, retrospective study, the healthcare services (e.g., outpatient visits, laboratory tests, imaging studies, dispensed medications, emergency department (ER) visits, hospitalizations, etc.) that were delivered at a university-affiliated tertiary center in Riyadh, Saudi Arabia, were retrospectively captured from the electronic medical records (EMRs) of patients with SCD who had at least 12 months of complete data. The data collection took place between 21 November 2023 and 17 June 2024. Patients who were seen only once for a medical consultation and had follow-up periods shorter than 12 months were not included in the analysis. Micro-costing was conducted to assign every service provided with a monetary value based on the prices for healthcare services published by the Saudi Council of Health Insurance (CCHI) [15]. Prescription medication prices were retrieved from the Saudi Food and Drug Authority website [16]. This study was approved by the research ethics committee at the College of Medicine at King Saud University. No personal identifiers were collected, such as names, medical record numbers, addresses, phone numbers, or national ID numbers. This study adhered to the research ethics principles of the Declaration of Helsinki [17], and this study was conducted from the perspective of public healthcare payers in Saudi Arabia.

### 2.2. Statistical Analyses

All of the collected data were anonymized to safeguard the patients’ privacy. The data collection was performed using Microsoft Excel (version 2019, Microsoft, Redmond, WA, USA). Descriptive statistics, including frequencies, percentages, means, and 95% confidence limits for the mean, were employed to present the baseline characteristics of the patients and the costs associated with the various categories of healthcare services. To investigate predictors and calculate the adjusted overall mean annual cost per patient, a generalized linear model (GLM) with a gamma distribution was utilized, which is particularly suitable for analyzing healthcare expenditures due to the heteroskedasticity often observed in healthcare cost data [18]. Various predictors of the direct annual medical costs of sickle cell disease (SCD), such as the frequency of lab and imaging tests, physician fees, blood transfusions, ER visits, hospitalizations, surgeries, rates of vaso-occlusive crises (VOCs), duration of illness, gender, and age, were included in the model based on the existing literature [19]. The cost data are presented in US dollars, using an exchange rate of 3.75 Saudi Riyals (SAR) per dollar. All of the statistical analyses were conducted using SAS^®^ version 9.4 (SAS^®^ Institute, Cary, NC, USA).

## 3. Results

Out of 156 EMRs for patients with SCD, 100 patients fit the inclusion criteria and were included in the analysis. Most of the patients (59%) were 10 or younger, and 53% were male. Most of the patients (97%) had the hemoglobin SS (HbSS) genotype, and 3% had HbS β-thalassemia. Only 6% of the patients had other comorbidities, such as hypertension, asthma, seizure, stroke, and hypothyroidism. In the past 12 months of patient follow-up, 44% had experienced at least one VOC. Moreover, 21% of the patients had undergone surgical procedures, such as cholecystectomy and splenectomy. Additionally, 59% of the patients had been hospitalized in the past 12 months at least once, and 28% had been hospitalized three times or more. Patients who had received at least one blood transfusion represented the majority (61%), and 33% had received three blood transfusions or more, as shown in Table 1.

**Figure 1:**
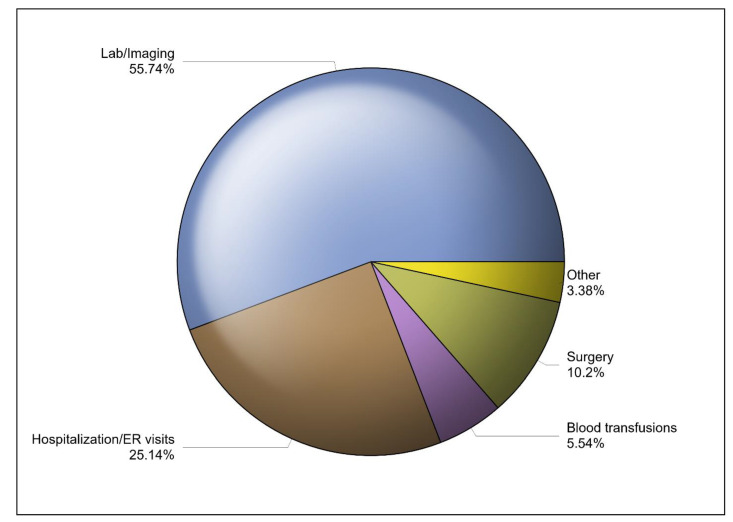
The percentages of different classes of direct medical costs for SCD management.

The regression estimates of the different variables that could influence the overall annual medical costs are shown in Table 2. The frequency of lab (β = 0.0617; 95% CI [0.0461–0.0779]; *p* < 0.0001) and imaging tests (β = 0.0617; 95% CI [0.0461–0.0779]; *p* < 0.0001) significantly predicts higher annual medical costs. Additionally, longer hospital stays (β = 0.0083; 95% CI [0.0020–0.0150]; *p* = 0.0110), frequent ER visits (β = 0.0609; 95% CI [0.0372–0.0850]; *p* < 0.0001), surgical procedures (β = 0.5023; 95% CI [0.3576–0.6470]; *p* < 0.0001), frequent blood transfusions (β = 0.0414; 95% CI [0.0135–0.0692]; *p* = 0.0036), and the number of prescription medications (β = 0.0007; 95% CI [0.0002–0.0013]; *p* = 0.0091) were associated with higher annual medical costs. Lab and imaging tests represented approximately 56% of the overall annual medical costs, followed by those of ER visits and hospitalization (25.14%), surgical procedures (10.2%), blood transfusions (5.54%), and others (3.38%), as shown in Figure 1. The mean annual cost per patient for each category of medical services is shown in Table 3. The mean annual medical cost per patient was estimated to be USD 26,626.45 [95% CI: USD 22,716.89–USD 30,536.0]. However, the adjusted mean annual medical cost per patient, which was controlled for age, gender, duration of illness, frequency of lab and imaging tests, surgical procedures, hospitalizations and length of stay, number of prescription medications, rates of VOCs, and rates of blood transfusions, was estimated to be USD 14,604.72 [95% CI: USD 10,943.49–USD 19,525.96].

The mean annual direct medical cost per patient for those who had undergone surgery was USD 44,559.70 (95% CI: USD 33,706.79–USD 55,412.61) compared to USD 22,419.88 [95% CI: USD 18,772.82–USD 26,066.95] for those who had not undergone surgery. Those who had been hospitalized in the past 12 months had a mean annual direct medical cost per patient of USD 35,584.94 [95% CI: USD 30,358.87–USD 40,811.01] compared to USD 13,734.96 in the opposite case [95% CI: USD 10,757.40–USD 16,712.51]. Patients who had paid at least one visit to the ER in the past 12 months had a mean annual direct medical cost per patient of USD 30,581.36 [95% CI: USD 26,030.35–USD 35,132.36] compared to USD 16,943.73 in the opposite case [95% CI: USD 10,228.65–USD 23,658.82]. Those who had undergone at least one RBC exchange had a mean annual direct medical cost per patient of USD 33,339.65 [95% CI: USD 27,815.50–USD 38,863.80] compared to USD 16,126.31 in the opposite case [95% CI: USD 13,028.38–USD 19,224.24]. The mean annual direct medical cost per patient across the gender and age categories is shown in Table 4.

## 4. Discussion

The burden of SCD is enormous and impacts different aspects for affected individuals, their families, and societies in different ways [12,14,19,20]. Despite the high prevalence and incidence rates of SCD in Saudi Arabia [21], no study has yet estimated the direct medical costs associated with this burdensome illness using real-world data, which makes this study timely. The mean annual direct medical costs associated with SCD management per patient are approximately USD 26,626, which is 20% higher than the estimated annual direct medical cost per patient from a recently published study that used self-reported measures to estimate the annual direct medical costs associated with SCD in Saudi Arabia [14]. However, due to the significant variation in the rates of healthcare service utilization among the study sample, the adjusted annual direct medical cost per patient was estimated to be USD 14,605, with wide confidence limits, ranging from USD 10,943 to USD 19,526. If these adjusted figures for the annual direct medical cost per patient were to be used to estimate the total annual direct medical costs, this would result in a mean total annual direct medical cost of USD 1,379,515,275 [95% CI: USD 1,033,621,065–USD 1,844,328,330] assuming that there are 94,455 SCD patients in Saudi Arabia, as stated in a previously published research abstract based on expert opinion [13]. This figure is huge considering the relatively small population of Saudi Arabia compared to that of the United States, which has an estimated burden of illness from the payer and patient perspectives of USD 2.98 billion per year [22]. This underscores the need to pay more attention to this unfortunately prevalent condition in certain geographic regions of Saudi Arabia where the rates of consanguineous marriages are high [8,21]. Genetic counseling and disease awareness campaigns to raise public awareness of this burdensome and debilitating health condition and its dire consequences are highly needed [23].

This study identified several drivers of direct medical costs among SCD patients. For example, the mean annual direct medical cost per patient for patients who underwent surgical procedures, such as cholecystectomy and splenectomy, was USD 44,559.70, which was 67.35% higher than the overall mean annual direct medical cost per patient. Moreover, patients who had been hospitalized in the past 12 months had a 33.65% higher mean annual direct medical cost per patient than the overall mean annual direct medical cost per patient. Additionally, patients who had visited emergency department services in the past 12 months had, on average, 14.85% higher costs than the overall mean annual direct medical cost per patient. Furthermore, patients who had received a blood transfusion in the past 12 months had, on average, a 25.21% higher cost than the overall mean annual direct medical cost per patient. These complications of SCD have been documented in several studies and have been shown to inflate its direct medical costs [12,13,19,20,21]. Although male patients with SCD have been shown to be higher utilizers of healthcare services in some studies, which was also found as well in this study, this was not statistically significant [24]. Another interesting finding in this study was the high utilization rates of laboratory and imaging tests among SCD patients in different settings (e.g., outpatient, inpatient, and ERs), which contributed to the largest segment of the annual direct medical cost.

Although this was the first study to utilize real-world evidence to estimate the direct medical costs associated with the management of SCD, it has several limitations that must be acknowledged. First, this study included only 100 patients from a single center, limiting its findings’ generalizability. Secondly, information bias cannot be ruled out since the study retrieved data from EMRs [25]. Moreover, patients with no follow-up visits were excluded, which may have resulted in a lower average cost. Additionally, this study was conducted from the payer’s perspective and did not include the patients’ perspective or informal care costs [26]. Moreover, this study was conducted from public payers’ perspective, which represents more than 60% of the healthcare provision in Saudi Arabia, and did not include private payers’ perspective, mainly due to the lack of healthcare cost data for this sector [27].

## 5. Conclusions

The direct medical costs related to the management of sickle cell disease (SCD) in Saudi Arabia are significantly high, underscoring the urgent need for the adoption and implementation of diverse preventative strategies, as well as advanced treatment options. Innovative therapies, particularly gene therapies, should be thoroughly assessed not only in terms of their clinical efficacy but also their potential to enhance the health-related quality of life (HRQoL) of patients suffering from SCD. This assessment should include metrics that evaluate improvements in daily functioning, pain management, and overall wellbeing. Moreover, it is crucial that future research examine both the direct and indirect costs associated with SCD from a comprehensive societal perspective. This entails employing nationally representative data to capture the full economic impact of the disease, including healthcare expenditures, the loss of productivity, and the costs borne by families and caregivers affected by SCD. Such studies could provide valuable insights that would inform health policy decisions, resource allocation, and the development of effective public health initiatives aimed at reducing the burden of SCD in the Saudi population.

## Figures and Tables

**Table 1:** Patients’ baseline characteristics (N = 100).

Characteristic	Frequency (%)
Gender	
Male	53 (53)
Female	47 (47)
Age	
<5 yrs.	18 (18)
5–10 yrs.	41 (41)
11–20 yrs.	37 (37)
≥21 yrs.	4 (4)
Duration of illness	
1–6 yrs.	35 (35)
7–10 yrs.	23 (23)
>10 yrs.	42 (42)
Genotype	
HbSS	97 (97)
HbS β-thalassemia	3 (3)
Patients with other chronic health conditions	6 (6)
Vaso-occlusive crisis in the past 12 months	
None	55 (55)
1–2	33 (33)
≥3	12 (12)
Surgical procedures in the past 12 months (e.g., tonsillectomy, splenectomy, cholecystectomy)	21 (21)
Hospitalization in the past 12 months	
None	41 (41)
1–2	31 (31)
≥3	28 (28)
Automated RBC exchange in the past 12 months	
None	39 (39)
1–2	28 (28)
3–4	22 (22)
≥5	11 (11)

**Table 2:** Generalized linear regression model with gamma distribution for the relationship between overall healthcare costs and different variables.

Variable	Estimate	95% Confidence Limits		*p*-Value
		Lower	Upper	
Age	−0.1412	−0.2836	0.0026	0.0509
Duration of illness	0.0453	−0.0486	0.1389	0.3389
Gender (male versus female)	0.0262	−0.0910	0.1432	0.6583
Frequency of lab tests	0.0617	0.0461	0.0779	<0.0001 *
Frequency of imaging studies	0.0001	0.0000	0.0002	0.0026 *
Length of hospital stay	0.0083	0.0020	0.0150	0.0110 *
Rate of emergency department visits	0.0609	0.0372	0.0850	<0.0001 *
Surgery	0.5023	0.3576	0.6470	<0.0001 *
Number of prescription medications	0.0007	0.0002	0.0013	0.0091 *
Rate of VOCs	0.0451	−0.0047	0.0949	0.0761
Rate of blood transfusions	0.0414	0.0135	0.0692	0.0036 *

* *p*-value < 0.05.

**Table 3:** Annual direct medical costs of sickle cell disease.

Cost Category in US (USD)	Mean	95% Confidence Limits	
		Lower	Upper
Lab tests	USD 14,375.87	USD 12,408.79	USD 16,342.96
Imaging studies	USD 465.44	USD 271.24	USD 659.65
Pharmaceuticals	USD 214.61	USD 187.74	USD 241.49
Inpatient admission	USD 4941.23	USD 3208.85	USD 6673.61
Surgery	USD 14,295.33	USD 10,320.75	USD 18,269.91
ER services	USD 5321.91	USD 4378.21	USD 6265.61
Automated RBC exchange	USD 2418.17	USD 2008.70	USD 2827.64
Healthcare labor	USD 685.44	USD 576.35	USD 794.53
Overall cost	USD 26,626.45	USD 22,716.89	USD 30,536.00
Adjusted overall cost	USD 14,604.72	USD 10,943.49	USD 19,525.96

**Table 4:** Stratified annual overall cost per patient based on demographic and medical characteristics.

Variable	Mean	95% Confidence Limits	
		Lower	Upper
Gender			
Male	USD 29,196.17	USD 23,192.86	USD 35,199.48
Female	USD 23,728.68	USD 18,788.65	USD 28,668.71
Age			
<5 yrs.	USD 33,279.31	USD 23,529.56	USD 43,029.05
5–10 yrs.	USD 25,488.39	USD 20,057.14	USD 30,919.64
11–20 yrs.	USD 25,339.51	USD 17,811.85	USD 32,867.17
21–30 yrs.	USD 17,918.02	USD 14,382.91	USD 62,835.78
>30 yrs.	USD 22,597.58	USD 5497.42	USD 239,875.65
Surgery			
Underwent surgical procedures	USD 44,559.70	USD 33,706.79	USD 55,412.61
Did not undergo surgical procedures	USD 22,419.88	USD 18,772.82	USD 26,066.95
Hospitalization			
Hospitalized	USD 35,584.94	USD 30,358.87	USD 40,811.01
Non-hospitalized	USD 13,734.96	USD 10,757.40	USD 16,712.51
ER visit			
Yes	USD 30,581.36	USD 26,030.35	USD 35,132.36
No	USD 16,943.73	USD 10,228.65	USD 23,658.82
Automated RBC exchange			
Yes	USD 33,339.65	USD 27,815.50	USD 38,863.80
No	USD 16,126.31	USD 13,028.38	USD 19,224.24

## Data Availability

The data are available upon reasonable request from the corresponding author.
